# Factors contributing to 1-year dissatisfaction after total knee arthroplasty: a nomogram prediction model

**DOI:** 10.1186/s13018-022-03205-2

**Published:** 2022-07-28

**Authors:** Mieralimu Muertizha, XinTian Cai, Baochao Ji, Abudousaimi Aimaiti, Li Cao

**Affiliations:** 1 Department of Orthopedics, First Affiliated Hospital of Xinjiang Medical University, 137th South LiYuShan Road, Urumqi, 830054 Xinjiang China; 2 Xinjiang Medical University Urumqi, People’s Republic of China, 137th South LiYuShan Road, Urumqi, Xinjiang China

**Keywords:** Total knee arthroplasty, Patient satisfaction, Nomogram, Coronal alignment

## Abstract

**Background:**

Identifying risk factors and early intervention are critical for improving the satisfaction rate of total knee arthroplasty (TKA). Our study aimed to identify patient-specific variables and establish a nomogram model to predict dissatisfaction at 1 year after TKA.

**Methods:**

This prospective cohort study involved 208 consecutive primary TKA patients with end-stage arthritis who completed self-reported measures preoperatively and at 1 year postoperatively. All participants were randomized into a training cohort (*n* = 154) and validation cohort (*n* = 54). Multiple regression models with preoperative and postoperative factors were used to establish the nomogram model for dissatisfaction at 1 year postoperatively. The least absolute shrinkage and selection operator method was used to screen the suitable and effective risk factors (demographic variables, preoperative variables, surgical variable, and postoperative variables) collected. These variables were compared between the satisfied and dissatisfied groups in the training cohort. The receiver operating characteristic (ROC) curve, calibration plot, and decision curve analysis were used to validate the discrimination, calibration, and clinical usefulness of the model. Results were evaluated by internal validation of the validation cohort.

**Results:**

The overall satisfaction rate 1 year after TKA was 77.8%. The nomogram prediction model included the following risk factors: gender; primary diagnosis; postoperative residual pain; poor postoperative range of motion; wound healing; and the rate of change in the degree of coronal lower limb alignment (hip–knee–ankle angle, HKA).The ROC curves of the training and validation cohorts were 0.9206 (95% confidence interval [CI], 0.8785–0.9627) and 0.9662 (0.9231, 1.0000) (95% CI, 0.9231, 1.0000), respectively. The Hosmer–Lemeshow test showed good calibration of the nomogram (training cohort, *p* = 0.218; validation cohort, *p* = 0.103).

**Conclusion:**

This study developed a prediction nomogram model based on partially modifiable risk factors for predicting dissatisfaction 1 year after TKA. This model demonstrated good discriminative capacity for identifying those at greatest risk for dissatisfaction and may help surgeons and patients identify and evaluate the risk factors for dissatisfaction and optimize TKA outcomes.

## Introduction

Total knee arthroplasty (TKA) is a successful treatment that provides pain relief and improves joint function in patients with end-stage arthritis [1]. TKA has been shown to be successful based on the results of a survival analysis and outcomes based on radiologic and clinical parameters. However, a mismatch may exist between surgeon-determined clinical records and patient-based satisfaction [2, 3]. Although substantial improvements in surgical technologies and prostheses have been achieved, the fact that one out of every five TKA patients remains dissatisfied after surgery has not changed during the past decades [4]. Patients want a knee that is “normal,” has restored premorbid function, and feels like a natural knee.

Dissatisfaction is the mental state or attitude of being discontent or displeased for a particular reason [5] TKA satisfaction is influenced by preoperative, surgical, and postoperative factors [1]. The most common reasons for dissatisfaction include residual pain, limited function, poor patient selection, poor surgical technique, malalignment of limbs, and postoperative complications [6, 7]. However, patients who have an appropriate component position, lower alignment, and well-functioning implants may also feel discomfort in the joint [8]. It is unclear why some patients are satisfied and others are not satisfied after TKA [9]. Even when TKA was performed by a team of surgeons with the same training, it was not possible to accurately predict patient dissatisfaction and patient-reported outcome measures postoperatively [10].

The identification of risk factors is an ongoing challenge for the clinical team and can help patients resolve their specific problems and optimize TKA outcomes [11]. The relative importance of preoperative predictors of postoperative dissatisfaction is not completely understood. Only a few publications have identified general predictors of patient satisfaction after TKA [12–15]. Knowledge of these predictors could help clinicians and patients participate in shared decision-making and support the optimal treatment decision [16].

A nomogram is a graphical description of a statistical model. It is useful for integrating potential risk factors to predict the probability of a clinical event occurring for an individual [17] and has been widely used for tumor prognoses [18] and heart diseases [19]. However, to our knowledge, there are no relevant studies of predicting dissatisfaction after TKA using a nomogram. After consulting the literature and reviewing studies of TKA satisfaction, we analyzed the most common factors affecting patient dissatisfaction and determined its risk factors. We utilized patient-derived data (demographic, preoperative, postoperative) and surgeon-derived data (surgical records) from a prospective patient cohort and created a nomogram prediction model. We evaluated the effectiveness of the nomogram and whether it could allow surgeons to improve patient selection and individualize procedures to improve patient satisfaction after TKA.

## Materials and methods

### Study design and patients

The hospital research and ethics committee approved the study protocol. From January 2017 to December 2019, 574 patients who underwent TKA for knee arthritis at our department were recruited for this study; 208 were finally included in our analyses. The inclusion criteria were primary TKA for arthritis (primary osteoarthritis [OA], rheumatoid arthritis [RA], and traumatic osteoarthritis), posterior stabilized rotating platform without patellar replacement, devices implanted using a ligament balancing technique, able to participate in the evaluation program, and completion of all study procedures and follow-up visits. The exclusion criteria were as follows: huge bony defects; history of a fracture that resulted in a deformity after healing that might change the bony alignment, such as high tibial or distal femoral osteotomies; partial knee replacement (unicompartmental, bicompartmental, or patellofemoral joint replacement); patellectomy; arthroscopy within 1 year; use of blocks, long stems, or special implants, such as constrained condylar knee implants; juvenile rheumatoid or psoriatic arthritis; systemic lupus erythematosus; significant neurological or musculoskeletal disorder or disease that may adversely affect gait or function recovery; lower back pain and pain in other joints; complications related to TKA within 1 year postoperatively; readmission for TKA complications, such as pulmonary embolism, hospital-acquired urinary tract infection, delayed wound healing, suture abscess, wound dehiscence; and the need for manipulation under anesthesia during the postoperative course because of arthrofibrosis, periprosthetic joint infection, recurvatum instability requiring revision, and prosthesis fracture.

Candidate variables were prospectively collected preoperatively and included age [20], gender [21], body mass index (BMI) [22], primary diagnosis, comorbidities [23], degree of anxiety [24], education level [25], marital status [25], smoking status [26], radiographic degree of OA in the femorotibial joint using the Kellgren–Lawrence scale [27] and coronal alignment of the lower limb (hip–knee–ankle [HKA] angle) measured by weight-bearing long-length radiographs [28]. Perioperative variables were the use of a tourniquet, anesthesia methods, local infiltration analgesia, and cosmetic closure. Postoperative variables included the range of extension, range of flexion, degree of pain, any other type of noise resulting from knee movement, and the postoperative coronal HKA angle.

A standard perioperative prophylactic antibiotic protocol was used for all patients. The type of anesthesia (general anesthesia or combined spinal–epidural anesthesia) was chosen based on the patient’s medical condition, concomitant spinal pathology, and vascular condition of the lower limbs. All patients included in this study underwent the same surgical technique for TKA. The same senior surgeon performed all surgical procedures. The standard surgical procedure for all patients included the medial parapatellar approach after anesthesia was administered. The ATTUNE® Knee System (DePuy Synthes, Warsaw, IN) or posterior stabilized rotating platform was implanted using a ligament balancing technique, and physical therapy was initiated on the first postoperative day for all patients. The decision to perform cosmetic suturing was based on the suturing difficulty.

### Self-reported measures

The Knee Society Score (KSS, 2011) is a comprehensive and validated measurement determined based on a clinician-reported objective subscale (not further evaluated in this study) and patient-reported subscales (including symptoms, satisfaction, and functional activity subscale). The higher the score, the better the outcomes of all subscales [29] The KSS takes into consideration patient reports of both expectations and satisfaction [30]. One year after TKA, we measured patient satisfaction by asking an overall satisfaction assessment question based on the 2011 KSS patient satisfaction subscale. As Van Onsem indicated [14], the satisfaction subscale consists of five items. Each item is scored using a scale from 0 to 5 points, resulting in a maximum KSS of 40; a score ≥ 20 indicated that the patient was satisfied and a score < 20 indicated that the patient was dissatisfied [31]. We adopted the same criteria. Finally, if patients underwent TKA multiple times, then we asked them to report the most recent surgery.

The pain degree (EQ-5L: pain) was rated as follows: I have no pain or discomfort = 0 points; I have slight pain or discomfort = 1 point; I have moderate pain or discomfort = 2 points; I have severe pain or discomfort = 3 points; and I have extreme pain or discomfort = 4 points [32].

Anxiety or degree of depression (EQ-5L: anxiety) was rated as follows [31]: I am not anxious or depressed = 1 point; I am slightly anxious or depressed = 2 points; I am moderately anxious or depressed = 3 points; I am severely anxious or depressed = 4 points; and I am extremely anxious or depressed = 5 points.

### Statistical analyses

Statistical analyses were performed using SPSS (version 23.0; IBM, Armonk, NY, USA) and R 4.0.2 (R Foundation for Statistical Computing, Vienna, Austria). Categorical variables were evaluated using the chi-square test. Continuous variables of the two groups were evaluated using the independent Student’s t test or Mann–Whitney U test. Univariate and multivariate logistic regression analyses were performed to identify the independent predictors of postoperative dissatisfaction. The data of the training cohort were analyzed using the least absolute shrinkage and selection operator (LASSO) method to screen suitable and effective risk factors for dissatisfaction. The results of univariate and multivariate regression analyses were visualized using forest plots. Finally, the results of the logistic regression analysis were used to construct a nomogram prediction model. The receiver operating characteristic (ROC) curve was used to quantify the discrimination performance of the nomogram, and calibration curves and the Hosmer–Lemeshow test were used to evaluate the calibration of the nomogram. A decision curve analysis was performed to determine the clinical applications of the risk prediction model. Bootstraps for 1000 resamples were performed using the ROC curve, calibration curve, and decision curve analysis to reduce the overfitting deviation. All statistical tests were two-sided, and P < 0.05 was considered significant.

## Results

**Fig. 1:**
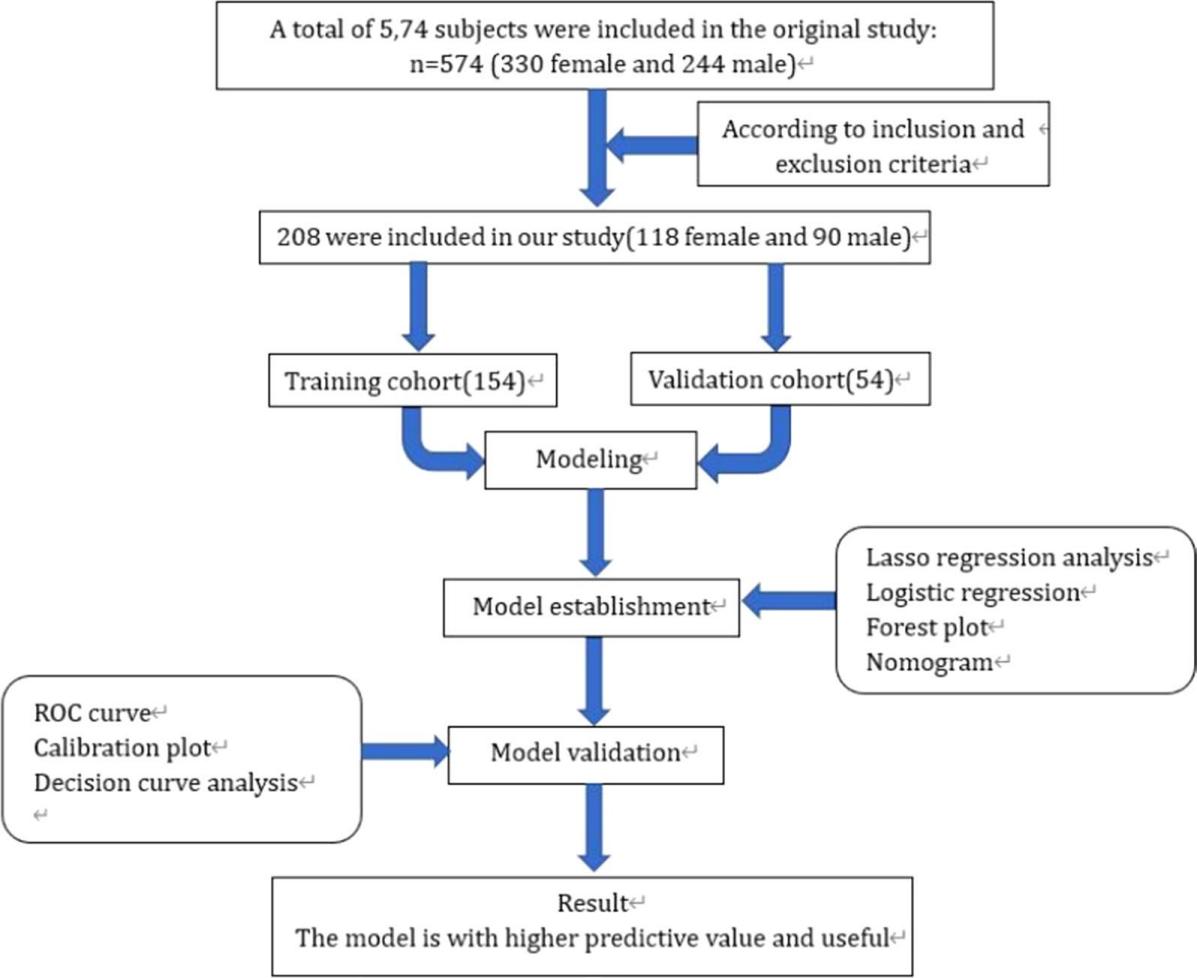
Flowchart illustration of study design

**Fig. 2:**
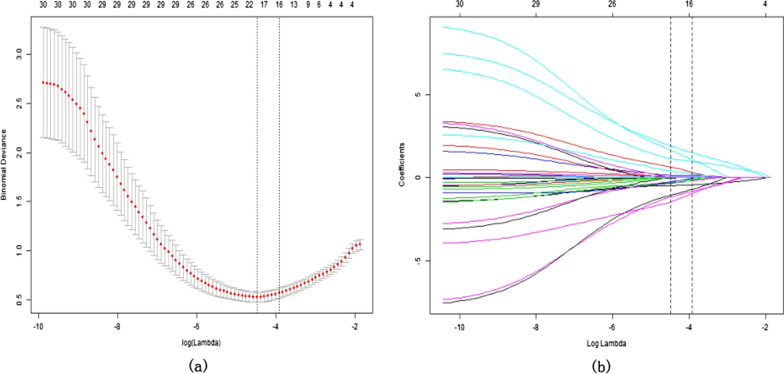
Demographic and clinical feature selection using the LASSO regression model. **a** Tenfold cross-validated error (first vertical line equals the minimum error, whereas the second vertical line shows the cross-validated error within 1 standard error of the minimum). **b** LASSO coefficient profiles of all the clinical features. A coefficient profile plot was produced against the log (lambda) sequence. Each of the different colored curves in the figure represents the trajectory of each independent variable coefficient. The vertical coordinate is the value of the coefficient, the lower horizontal coordinate is log (lambda), and the upper horizontal coordinate is the number of nonzero coefficients in the model. LASSO: least absolute shrinkage and selection operator; SE: standard error

A total of 208 participants were included in this study. For external validation, the R package was used to randomly divide them into the training cohort (*n* = 154) and the validation cohort (*n* = 54), consistent with a theoretical ratio of 3:1. A flow diagram of the study design is shown in Fig. 1. The baseline characteristics of the training and validation cohorts are summarized in Table 1. The overall incidence of satisfaction was 77.8% (162/208). For the training and validation cohorts, the rates of dissatisfaction were 121 (78.57%) and 41 (75.93%), respectively. No significant differences were observed between the two cohorts. Thirty potential risk factors were selected from the demographic and clinical characteristics and analyzed by LASSO regression (Fig. 2a and b). Nonzero characteristic variables were selected based on the statistical approach of the LASSO regression model. Therefore, the number of potential variables was reduced to six: gender, primary diagnosis, postoperative residual pain, poor postoperative range of motion, wound healing, and the rate of change in the degree of coronal lower limb alignment (HKA angle). Table 2 shows the specific coefficients corresponding to the variables.

**Table 1:** Demographic and clinical characteristics of the study population in the training and validation cohorts (*n* = 210)

Variables (*n*(%))	Total	Training cohort	Validation cohort	P value
Number of patients	208	154	54	
Age (Mean + SD)(year)		69.29 ± 7.11	69.29 ± 7.11	0.813
Gender				0.072
Male	90	61 (39.61%)	29 (53.70%)	
BMI (Kg/m^2^)		28.20 ± 3.42	28.20 ± 3.50	0.994
Primary diagnosis				0.106
Osteoarthritis (OA)	167	122 (79.22%)	45 (83.33%)	
Rheumatoid arthritis (RA)	33	28 (18.18%)	5 (9.26%)	
Post-traumatic arthritis	8	4 (2.60%)	4 (7.41%)	
Hypertension	120	94 (61.04%)	26 (48.15%)	0.099
Heart disease	100	72 (46.75%)	28 (51.85%)	0.519
Diabetes mellitus	107	97 (62.99%)	30 (55.56%)	0.335
Urinary system diseases	72	55 (35.71%)	17 (31.48%)	0.574
Respiratory diseases	102	79 (51.30%)	23 (42.59%)	0.271
Anxiety/depression				0.518
Not anxious or depressed	114	81 (52.60%)	33 (61.11%)	
Slightly anxious or depressed	72	55 (35.71%)	17 (31.48%)	
Slightly anxious or depressed	18	14 (9.09%)	4 (7.41%)	
Severely anxious or depressed	4	4 (2.60%)	0 (0.00%)	
Extremely anxious or depressed	0	0 (0.00%)	0 (0.00%)	
Education degree				0.177
Less than high school	79	62 (40.26%)	17 (31.48%)	
High school	71	47 (30.52%)	24 (44.44%)	
College degree and above	58	45 (29.22%)	13 (24.07%)	
Smoke	144	108 (70.13%)	36 (66.67%)	0.635
Marital status				0.791
Married	163	119 (77.27%)	44 (81.48%)	
Divorced/seperated	24	19 (12.34%)	5 (9.26%)	
Widowed	21	16 (10.39%)	5 (9.26%)	
Oral opioids before operation				0.614
Never	64	45 (29.22%)	19 (35.19%)	
Less than 3 months	93	69 (44.81%)	24 (44.44%)	
More than 3 months	51	40 (25.97%)	11 (20.37%)	
Anesthesia mode				0.046
General	124	98 (63.64%)	26 (48.15%)	
Epidural block	84	56 (36.36%)	28 (51.85%)	
Local anesthetic infltration	149	110 (71.43%)	39 (72.22%)	0.911149
Kellgren and Lawrence grade				0.266
I grade	0	0(0%)	0(0%)	
II grade	5	3 (1.95%)	2 (3.70%)	
III grade	84	58 (37.66%)	26 (48.15%)	
IV grade	119	93 (60.39%)	26 (48.15%)	
Tourniquet use	181	128 (83.12%)	43 (79.63%)	0.564
Cosmetic closure	159	119 (77.27%)	40 (74.07%)	0.634
Postoperative pain				0.821
No pain or discomfort	57	41 (26.62%)	16 (29.63%)	
Slight pain or discomfort	99	72 (46.75%)	27 (50.00%)	
Moderate pain or discomfort	44	35 (22.73%)	9 (16.67%)	
Severe pain or discomfort	8	6 (3.90%)	2 (3.70%)	
Extreme pain or discomfort		0(0%)	0(0%)	
Postoperative ROM				0.663
Less than 90°		0(0%)	0(0%)	
90–100°	10	7 (4.55%)	3 (5.56%)	
100–110°	30	21 (13.64%)	9 (16.67%)	
110–120°	47	38 (24.68%)	9 (16.67%)	
More than 120°	121	88 (57.14%)	33 (61.11%)	
Postoperative knee extension				0.423
Completely extended	150	110 (71.43%)	40 (74.07%)	
Lack of less than 5°	48	38 (24.68%)	10 (18.52%)	
Lack of 5–10°	8	6 (3.90%)	4 (7.41%)	
More than 10°		0(0%)	0(0%)	
Patella clicks				0.843
Never	129	95 (61.69%)	34 (62.96%)	
Rarely	54	40 (25.97%)	14 (25.93%)	
Sometimes	23	18 (11.69%)	5 (9.26%)	
Often	2	1 (0.65%)	1 (1.85%)	
Always		0(0%)	0(0%)	
HKA before operation (°)		− 5.74 ± 7.99	− 6.52 ± 7.53	0.533
HKA under operation (°)		− 1.63 ± 3.01	− 2.35 ± 2.97	0.129
Correction degree of HKA 1 year after TKA (Mean + SD)(°)		5.74 ± 7.99	6.52 ± 7.53	0.533
Rate of change in the degree of coronal lower limb alignment after TKA (%)		40.14 ± 23.83	41.33 ± 19.04	0.740
Satisfaction at 1 year	162	121 (78.57%)	41 (75.93%)	0.687

**Table 2:** Coefficients and lambda.1se value of the LASSO regression based on the training cohort

Factors	Coefficients	Lambda.1se
Primary diagnosis	0.987	0.0198
Gender	− 0.943	
Cosmetic closure	− 0.816	
Postoperative ROM	− 0.431	
Postoperative pain	1.504	
Correction rate of HKA 1 year after TKA (%)	0.048	

### Univariate and multivariate Cox regression analyses of the training cohort

**Fig. 3:**
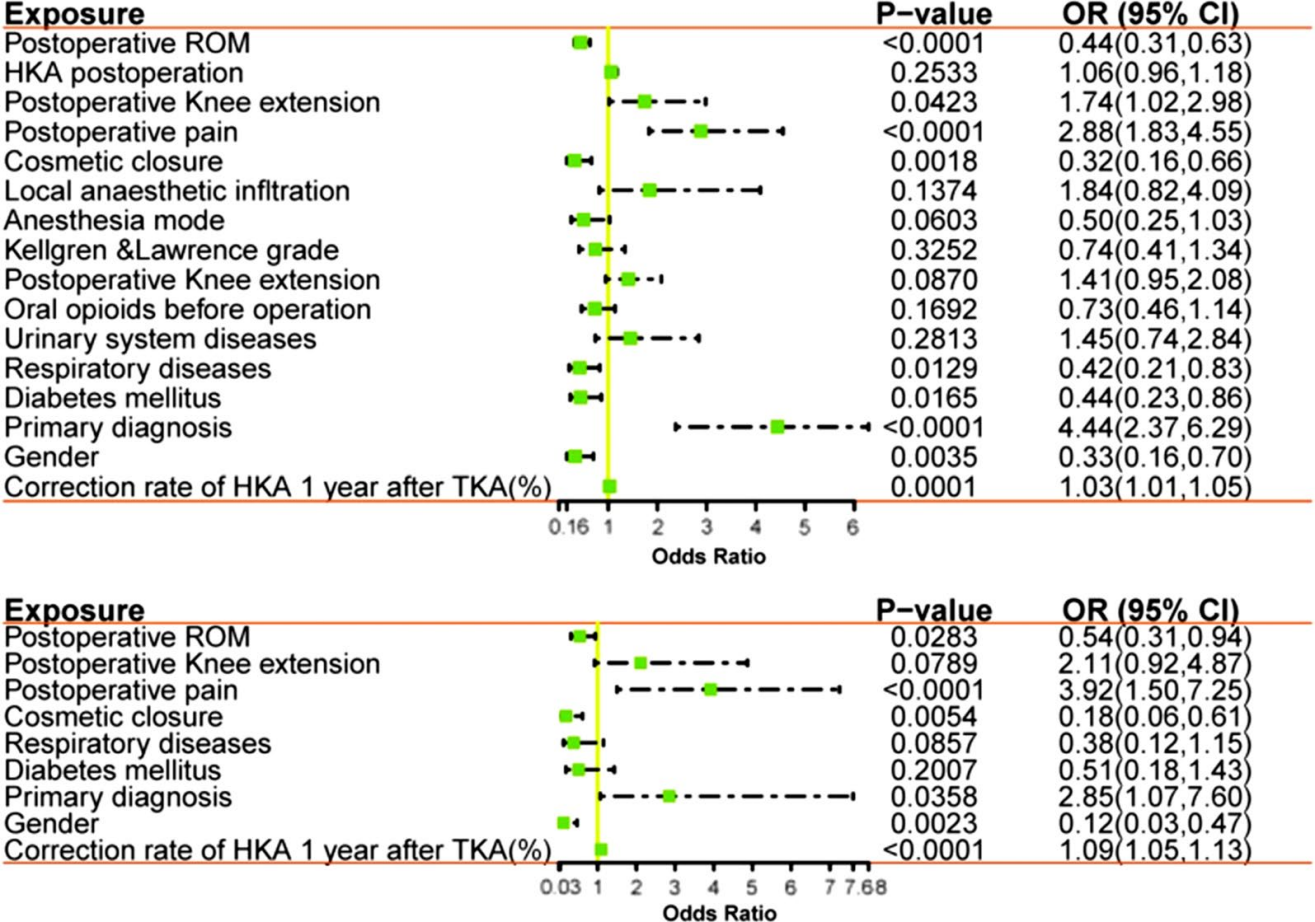
Forest plot of univariable and multivariable cox regression analysis for risk factors associated with dissatisfaction. HR: hazard ratio; CI: confidence interval

The univariate analysis (Fig. 3a) and multivariate Cox regression analysis (Fig. 3b) were performed for 154 TKA patients in the training cohort. The results showed that gender, primary diagnosis, postoperative residual pain, poor postoperative range of motion (ROM), wound healing, and the rate of change in the degree of coronal lower limb alignment (HKA angle) were considered independent predictors of dissatisfaction after TKA (*p* < 0.05).

### Development of the prediction model

**Fig. 4:**
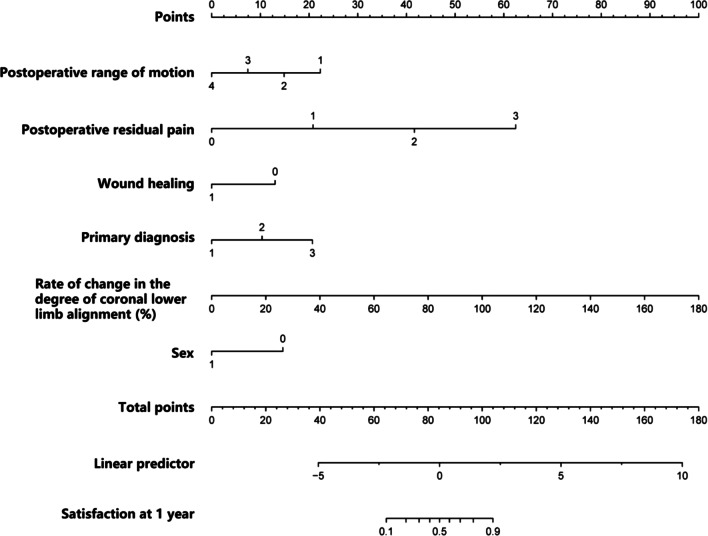
Nomogram for predicting the dissatisfaction risk of patients 1 year postoperatively. To use the nomogram, an individual patient’s value is located on each variable axis, and a line is drawn upward to determine the number of points received for each variable value. The scores for all variables are then added to obtain the total score, and a vertical line is drawn from the total points row to estimate the risk of dissatisfaction in TKA patients 1 year postoperatively at the lower line of the nomogram

We combined the six independent predictors into a predictive model and displayed them in the form of a nomogram (Fig. 4), which is a quantitative and convenient tool. To obtain the individualized risk of patient dissatisfaction 1 year after TKA, the point value for each variable was assessed; then, all values were summed to obtain the total score to determine the risk.

### Performance of the nomogram

**Fig. 5:**
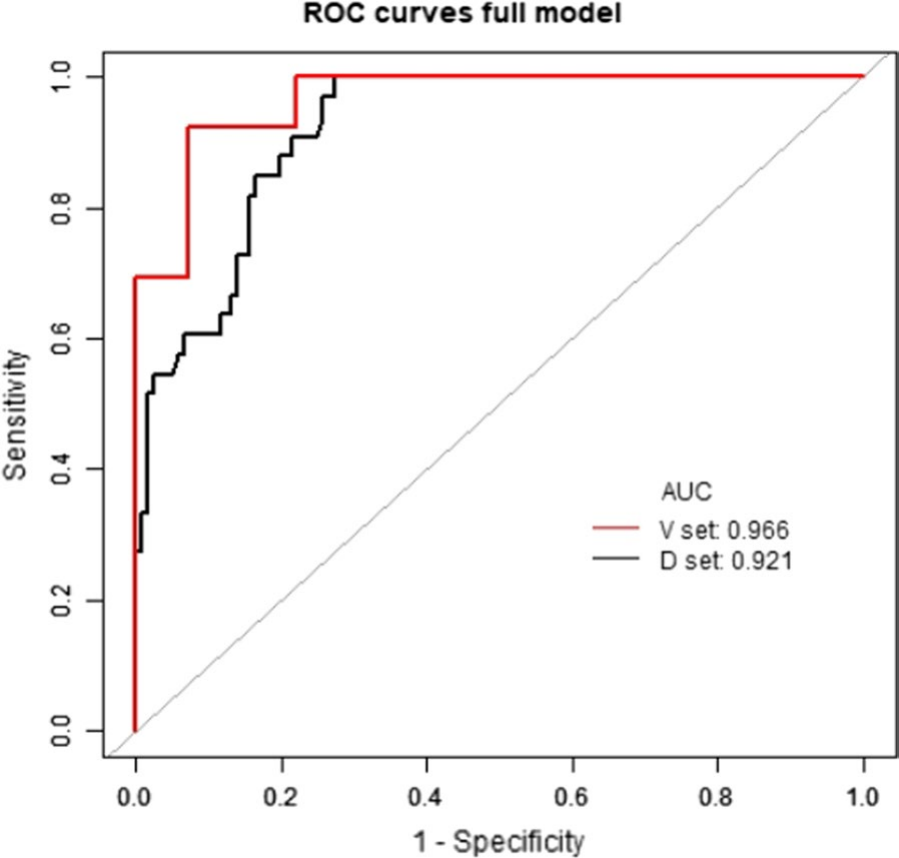
ROC curve of the nomogram in the training and validation cohort. ROC: receiver operating characteristic; AUC: area under the curve (bootstrap resampling times = 1000)

**Fig. 6:**
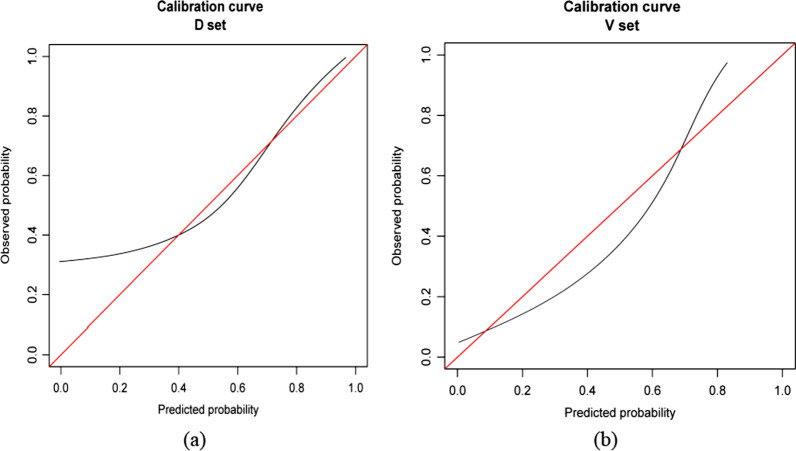
Calibration curves for the training and validation cohort models. **a** Calibration curve of the nomogram in the training cohort. **b** Calibration curve of the nomogram in the validation cohort. The red curve is a calibration curve corresponding to the actual situation. The blue curve represents the 95% CI range of the calibration curve (bootstrap resampling times = 1000)

**Fig. 7:**
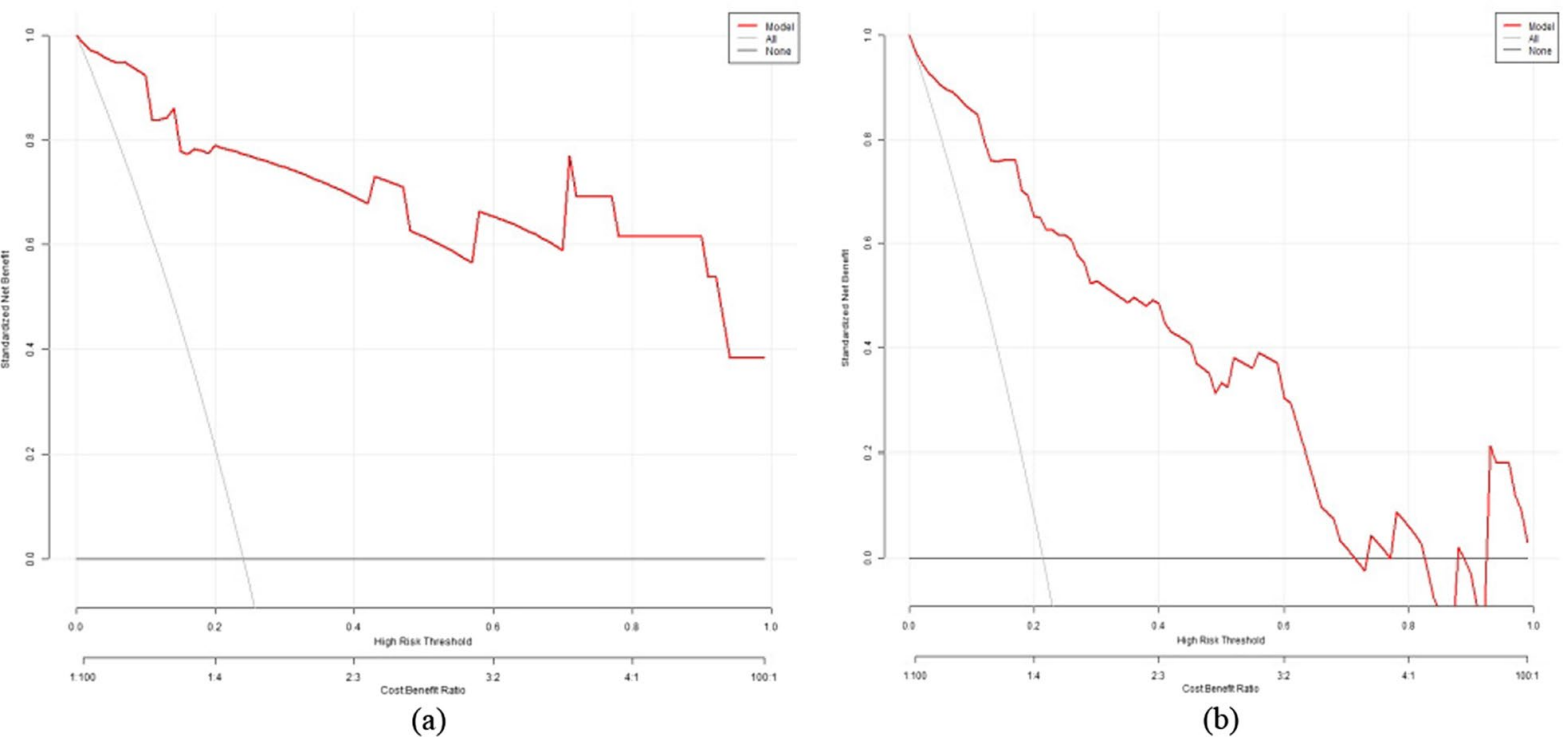
Decision curve analysis of the nomogram in the training (**a**) and validation cohorts (**b**). The *y* axis stands the net benefit. The *x* axis indicates the threshold probability. The red line represents the nomogram. The black line displays the net benefit of the strategy of treating no patients. The gray line displays the net benefit of the strategy of treating all patients (bootstrap resampling times = 1000)

The ROC curve was used to evaluate the discriminatory ability of the prediction model. The pooled area under the curve of the nomogram was 0.9206 (95% CI: 0.8785, 0.9627), with sensitivity and specificity of 100% and 72.73%, respectively, for the training cohort (Fig. 5a). For the validation cohort, the pooled area under the curve was 0.9662 (95% CI: 0.9231, 1.0000), with sensitivity and specificity of 92.31% and 92.68%, respectively (Fig. 5), indicating moderately good performance (Table 3). The calibration curve and Hosmer–Lemeshow test indicated that the prediction model showed good fit for both the training and validation cohorts (Fig. 6a and b). Furthermore, the Hosmer–Lemeshow test indicated good agreement between the predicted and actual probabilities for both the training and validation cohorts. The decision curve analysis of the training cohort (Fig. 7a) and validation cohort (Fig. 7b) indicated that the application of the model for TKA patients to predict the risk of dissatisfaction 1 year after surgery was more effective than intervention for all patients. Each patient was separated into a high-risk group or low-risk group according to a cutoff value of 50% predicted by the nomogram.

**Table 3:** ROC in the nomogram based on training cohort and validation cohort

	ROC (95% CI)	Sensitivity	Specificity	*n*
Training cohort	0.9206 (0.8785, 0.9627)	1.0000	0.7273	154
Validation cohort	0.9662 (0.9231, 1.0000)	0.9231	0.9268	52

### Clinical usefulness of the nomogram

The decision curve showed that it would be more accurate to use the nomogram in the development cohort to predict the risk of dissatisfaction when the risk threshold probability was between 1 and 99%, and in the validation cohort, when between 1 and 71% (Fig. 7a and b). We applied the nomogram in TKA patients as an example. The first patient had postoperative range of motion, postoperative pain, wound healing, primary diagnosis, rate of change in the degree of coronal lower limb alignment after TKA (%), the gender of the patients. These were between 90 and 100° (21.9 points), with moderate pain or discomfort (41.6 points), without cosmetic closure (12.9 points), with rheumatoid arthritis (RA) (10.3 points), (80%) (44.4 points), and a female (14.6 points), respectively. The total calculated nomogram score was 145.7, and the 1-year risk of dissatisfaction after TKA was more than 95%. This patient had a high risk of dissatisfaction 1 year after TKA. The second patient had postoperative range of motion, postoperative pain, wound healing, primary diagnosis, rate of change in the degree of coronal lower limb alignment after TKA (%), and the gender of the patients. There were between 110 and 120° (7.4 points), with slight pain or discomfort (20.8 points), with cosmetic closure (0 points), with osteoarthritis (OA) (0 points), (20%) (11.1 points), and a male (0 points), respectively. The total calculated nomogram score was 39.3, and the 1-year risk of dissatisfaction after TKA was less than 5%. This patient had a very low risk of dissatisfaction 1 year after TKA.

## Discussion

There are two main challenges when developing a useful predictive nomogram: choosing the right predictive factors and selecting those outcomes that are both predictable and useful measures of clinical satisfaction. The current study showed that the most important risk factors for predicting dissatisfaction were gender, primary knee diagnosis (traumatic knee arthritis), persistent pain after TKA, poor knee flexion, cosmetic closure, and the rate of change in the degree of coronal lower limb alignment. Our study aimed to develop and examine a predictive model of satisfaction for patients 1 year after TKA. According to the research, overall satisfaction is stable approximately 12 months after TKA [33]. Patient satisfaction is difficult to evaluate because there is no gold standard method of measurement. We used the 2011 KSS to define satisfaction after TKA because it is a highly useful instrument for comprehensively evaluating patients before and after TKA [30, 34, 35].

During our study, the rate of satisfaction was 77.6%, which is consistent with the results of other recent studies [1, 30]. We found that the most important factors that outweighed surgical factors (such as anesthesia type, use of a tourniquet) and other patient factors were age [20], BMI [22], marital status [25], comorbidities [23], depression or anxiety [24], smoking [26], education [25], and local infiltration analgesia [36]. However, age and BMI are controversial factors associated with TKA satisfaction. Giesinger et al. found that BMI had a negative impact on postoperative satisfaction scores [22]. Some studies have shown that age younger than 55 years is an independent predictor of functional recovery and patient satisfaction [20, 37]. In our study, there were only six patients younger than 55 years; therefore, we could not completely judge the difference between younger and older patients. It is possible that physiological age may be more predictive than chronological age because fitter and more active patients had higher expectations [38]. Although not statistically significant, it is likely that the evaluation of lower-risk tiers will result in a lower likelihood of dissatisfaction.

The key contributing variables in our model are in line with those reported by previous publications; for example, female patients, on average, were given higher doses of pain medication than male patients [39] and male patients, on average, tolerated higher muscle pressure pain than female patients (*p *= 0.003 and *p *= 0.02) [40]. Although some studies indicated that even though gender differences exist, knee and hip anatomy differences have been well accepted; however, these did not show a significant correlation with TKA satisfaction [21] Furthermore, no difference in the outcomes of patients with gender-specific knee arthroplasty compared to conventional arthroplasty was observed, and gender-specific TKA did not provide any benefit over other procedures [41]. Patients in our study with RA had lower satisfaction and some flexion deficits compared to OA patients postoperatively; however, these results were not consistent with those of other studies [42, 43]. This may have occurred because most RA patients in our study delayed surgery longer, there is a lack of systematic and standardized medical treatment for RA after TKA, and RA patients have other joint pain. We found that many RA patients have some degree of depression and lower baseline expectations than patients with OA [44]. There were eight traumatic knee patients in our study, and their functional recovery and satisfaction after TKA were lower than those of OA and RA patients. Few studies of the traumatic knee treated with TKA exist [45]. Therefore, we considered that this occurred because post-traumatic deformity and compromise of the soft tissue envelope influence the pain and functional outcomes of patients who undergo TKA for post-traumatic arthritis [46].

Residual pain seems to be the most prominent cause of dissatisfaction after TKA, with 7% to 34% of patients reporting unfavorable long-term residual pain [47]. However, the mechanism of residual pain after TKA is complicated, and it may be difficult to eliminate pain completely. Therefore, the identification of risk factors for high pain levels is essential to improving postoperative pain relief and rehabilitation to reduce the dissatisfaction rate. One study stated that patients with less severe OA were much less likely to achieve a patient-acceptable state of pain and function at 1 year after TKA, and that men were much less likely to achieve a patient-acceptable state of pain after TKA [48], surgeons should strongly consider delaying TKA for men with less severe OA, and they should counsel their patients regarding their expectations [48].

With the trend toward enhanced recovery after surgery, the method of skin closure has become increasingly important in joint replacement. We found that patients who underwent subcuticular cosmetic closure, particularly female patients, were more likely to be satisfied with their wound healing after TKA. Contrary to our opinion, Nepal et al. performed a randomized control trial and reported that subcuticular sutures and staples were comparable wound closure options relative to cosmetic outcomes, patient satisfaction, functional outcomes, and wound complication rates after TKA [49]. Concealed cosmetic closure [50] and Dermabond Prineo [51] may be effective modalities for skin closure after TKA that provide superior cosmetic healing with minimal complications, thereby leading to improved long-term patient satisfaction. However, studies have not provided a clear evidence-based answer regarding which closure method is optimal for TKA; therefore, more high-quality evidence-based studies are needed before the final conclusion can be determined [52].

We found that poor flexion may cause dissatisfaction after TKA because it limits basic activities, such as climbing and squatting, in daily life. The ROM also contributes to dissatisfaction after TKA. Common reasons for poor ROM are arthrofibrosis, stiffness, and contracture of the knee, which can lead to limitations in daily activities [53]. Numerous risk factors, such as genetics, post-traumatic knee [46, 54], poor preoperative ROM, and higher BMI, may cause patient dissatisfaction with postoperative ROM [55]. Surgical factors associated with poor ROM include malalignment and improper gap imbalance, and postoperative factors associated with dissatisfaction include poor rehabilitation therapy, decreased patient motivation, and pain management [53].

Our research highlighted that there was a large rate of change in the HKA angle after TKA, which may cause unfavorable feelings after surgery. In our study, preoperative planning pursued a neutral mechanical axis (180° ± 3°), and the distal femoral osteotomy line was created perpendicular to the femoral mechanical axis, which was measured using preoperative long-length radiographs. We found that patients with severe varus and valgus deformities preoperatively should not undergo single treatment to achieve neural alignment. Instead, we should consider creating a better flexion and extension gap, balanced mediolateral soft tissue tension strength, joint line obliquity, restoration of patellar tracking, and rotational axis of the femur–tibial joint. Similarly, restoring neutral alignment in these patients with constitutional varus may be unfavorable because more tissue release would likely be needed to achieve neutral alignment, thereby causing pain and discomfort after TKA [8]. Recent research has advocated that the aim of functional alignment should include implantation of components with minimal compromise of the soft tissue envelope by restoring the plane and obliquity of the nonarthritic joint during TKA [56].

The prediction model proposed in this study is advantageous because it was created based on modifiable clinical risk factors, thereby allowing for beneficial preoperative counseling and potential shifts toward nonsurgical management. Furthermore, it allows for objective risk modification before surgical intervention, better surgical decisions during surgery, and early intervention after TKA.

This study had several limitations. Because our research cohort was prospective, follow-up is ongoing and will be available in the future for analysis. Similarly, patient recruitment is ongoing; therefore, there is potential for a larger patient cohort in the future, possibly resulting in a more robust model. Second, because this nomogram is specific to TKA and our region, it may not be generalizable to other orthopedic populations. The patients were from a single institution; therefore, this predictive model lacks a prospective cohort for external validity. Despite the use of bootstrapping procedures to balance the capabilities of the prediction model, the diagnostic power may have been overestimated, indicating that the findings were overly optimistic. In our nomogram, we were using many categorical variables and it may have led to less robust prognostic risk prediction. We consider that it is mainly because most of the relevant variables collected in this study were categorical variables. Future studies are warranted to determine the extent of external validity; therefore, we encourage other institutions to validate this model using patient cohorts. Third, it is possible that the relative weights of some survey subcomponents may require adjustment. Finally, we did not control for other potentially confounding variables in our regression models, such as different implant designs and postoperative joint laxity. In the future, we aim to prospectively validate this study, assess whether the relative weights of subcomponents require adjustments, and determine what preoperative time period is necessary for patients to accomplish risk tier transition.

## Conclusion

For the training and validation cohorts, our nomogram had excellent prediction performance and excellent calibration curve consistency. The decision curve analysis illustrated the clinical application value of our nomogram. Additionally, the nomogram will be of great practical value because of its easily available parameters and may allow for improved TKA satisfaction.

## Data Availability

The datasets used and/or analyzed during the current study are available from the corresponding author on reasonable request.

## Abbreviations

BMI: Body mass index
HKA: Hip–knee–ankle
KSS: Knee society score
LASSO: Least absolute shrinkage and selection operator
OA: Osteoarthritis
RA: Rheumatoid arthritis
ROC: Receiver operating characteristic
ROM: Range of movement
TKA: Total knee arthroplasty
